# Combination of underwater and traction-assisted endoscopic submucosal dissection: the perfect wedding for a challenging case

**DOI:** 10.1055/a-2723-1869

**Published:** 2025-11-05

**Authors:** Said Al Alawi, Talat Bessissow, Stephen Tsoukas, Romina Ureña Campos, Yidan Lu, Yen-I Chen, Jérémie Jacques

**Affiliations:** 1 54473 Division of Gastroenterology and Hepatology, McGill University Health Centre, Montreal, Canada; 2 60301 Gastroenterology, Maisonneuve-Rosemont Hospital, Montreal, Canada; 3 Gastroenterology and Hepatology, CHU Dupuytren, Limoges, France

Various modifications have been introduced to enhance the effectiveness of endoscopic submucosal dissection (ESD), including tunneling, the pocket-creation method, traction-assisted techniques, hybrid ESD (combined with EMR), and, more recently, underwater ESD 1 2 . In this report, we present a case utilizing a combined approach of traction and underwater-assisted ESD.

A 72-year-old female presented with a 7 cm granular homogeneous lateral spreading tumor (LST) involving the cecum and extending deeply into the appendix in a patient without prior history of appendectomy ( Fig. 1 ). ESD was chosen due to both the size of the lesion and its extension into the appendix.

**Fig. 1:**
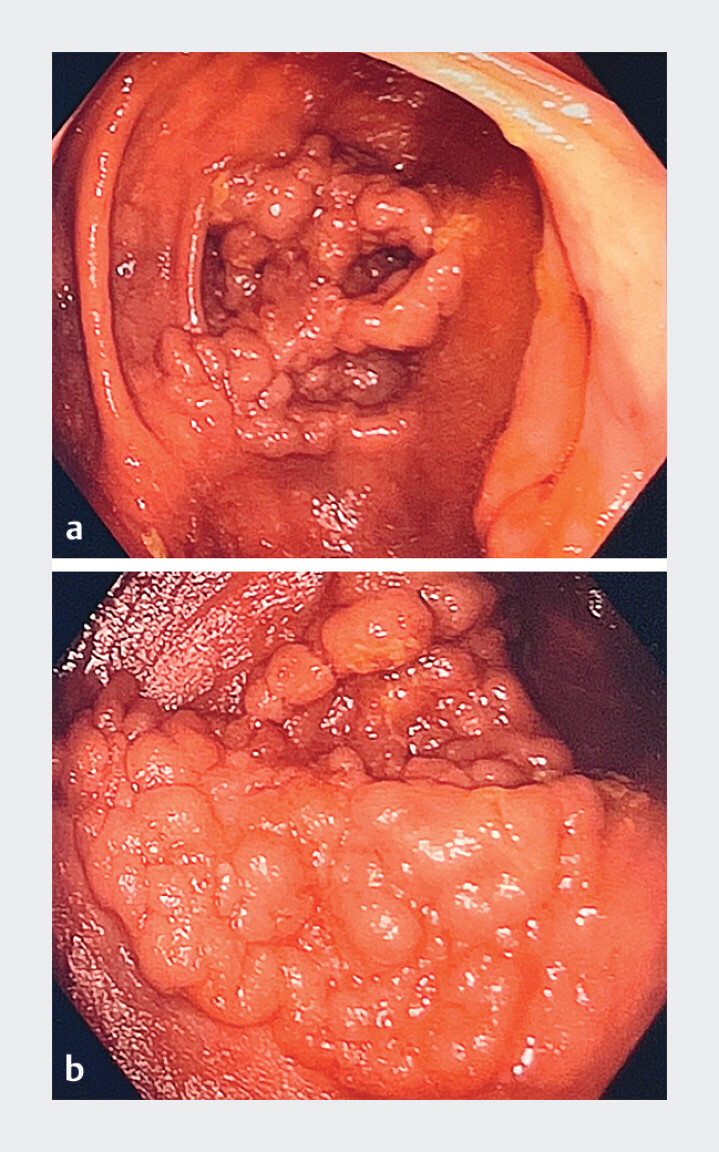
Endoscopy picture of a 7 cm granular homogeneous lateral spreading tumor (LST) involving the cecum and extending deeply into the appendix.

The procedure was initiated with underwater ESD under propofol deep sedation, achieving complete circumferential dissection around the appendix. After completion of the dissection except for the appendiceal portion, a traction device was placed in a reverse position on the appendix remnant within the lumen. This maneuver allowed the appendix to be pulled fully into the cecal lumen, as demonstrated in the procedural video, and facilitated en bloc resection ( Video 1 ). The 7 cm lesion was successfully resected within 90 minutes without perforation ( Fig. 2 and Fig. 3 ). Closure was performed selectively at the appendiceal scar site using a combination of Mantis clips and through-the-scope clips (TTC) to reduce the risk of delayed complications ( Fig. 3 ). Pathology revealed a tubulovillous adenoma with focal high-grade dysplasia and clear resection margins.

Combination of underwater and traction-assisted ESD for resection of a large cecal LST extending into the appendiceal lumen.Video 1

**Fig. 2:**
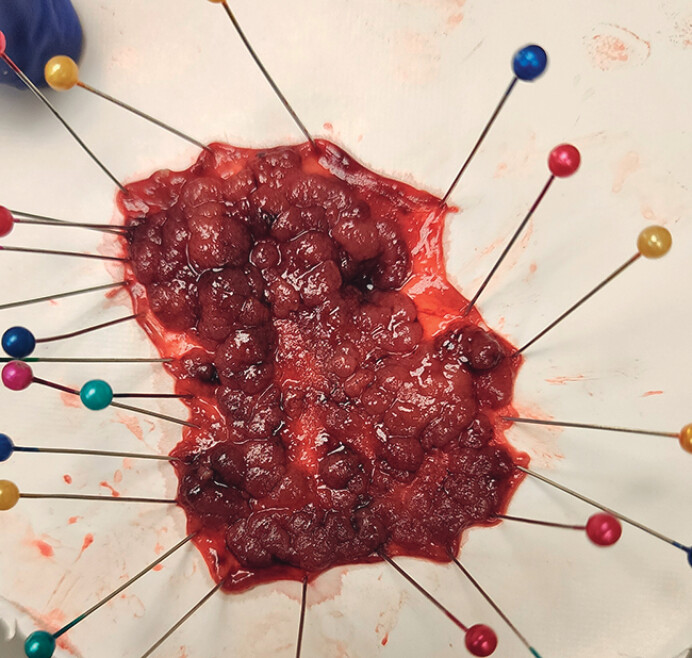
Picture of the endoscopic submucosal dissection (ESD) specimen of 7 cm LST.

**Fig. 3:**
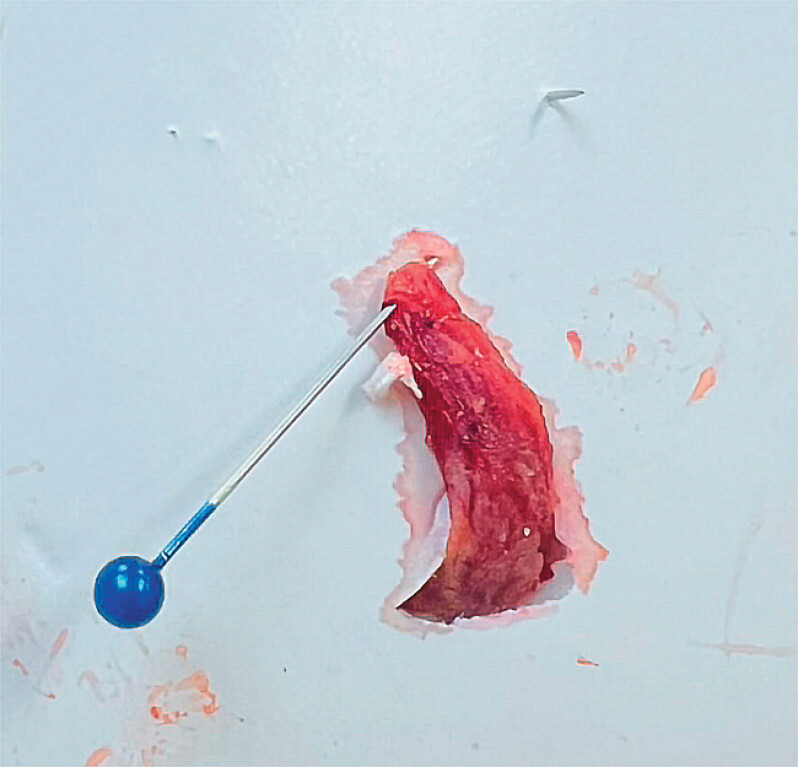
Picture of the endoscopically resected appendicular specimen.

This case highlights the value of combining underwater ESD and traction-assisted ESD as complementary strategies in technically challenging situations. Both techniques are increasingly recognized by endoscopists as practical tools that can be tailored together, with distinct advantages and limitations, to optimize outcomes in difficult resections 3 4 .

Endoscopy_UCTN_Code_TTT_1AQ_2AD_3AD

## Notes

Endoscopy E-Videos https://eref.thieme.de/e-videos
*E-Videos* is an open access online section of the journal *Endoscopy* , reporting on interesting cases and new techniques in gastroenterological endoscopy. All papers include a high-quality video and are published with a Creative Commons CC-BY license. Endoscopy E-Videos qualify for HINARI discounts and waivers and eligibility is automatically checked during the submission process. We grant 100% waivers to articles whose corresponding authors are based in Group A countries and 50% waivers to those who are based in Group B countries as classified by Research4Life (see: https://www.research4life.org/access/eligibility/ ). This section has its own submission website at https://mc.manuscriptcentral.com/e-videos .

## References

[1] Singh S Mohan BP Vinayek R Underwater versus conventional endoscopic submucosal dissection for colorectal lesions: systematic review and meta-analysis Gastrointestinal Endosc 2025 101 551 5.57E7 10.1016/j.gie.2024.10.029 39427993

[2] Yoshii S Hayashi Y Matsui T “Underwater” endoscopic submucosal dissection: a novel technique for complete resection of a rectal neuroendocrine tumor Endoscopy 2016 48 01 E67 E68 10.1055/s-0042-101855 26890547

[3] Tada N Tamai N Ito M Synergistic advantages of combining a traction device with underwater conditions for colonic endoscopic submucosal dissection Endoscopy 2025 57 01 E664 E666 40570921 10.1055/a-2598-5291 PMC12202114

[4] Hamada K Horikawa Y Shiwa Y Underwater and traction-assisted endoscopic submucosal dissection in the gastric fundus using a multibending endoscope Endoscopy 2022 55 01 E312 E313 10.1055/a-1974-9792 36513103 PMC9833949

